# Legacy of Recurrent Gene Flow and Evidence of Independent Evolution of ALS Resistance Mutations in Weedy Rice

**DOI:** 10.1111/eva.70342

**Published:** 2026-10-27

**Authors:** Guilherme Menegol Turra, Paula Sinigaglia Angonese, Eduardo Carlos Rudell, Vinícius Ferrari Tasca, Catarine Markus, Aldo Merotto Junior

**Affiliations:** ^1^ Crop Science Departament Federal University of Rio Grande Do Sul Porto Alegre RS Brazil

**Keywords:** ALS inhibitors, molecular markers, *Oryza sativa*, SNAP, target‐site resistance

## Abstract

Weedy rice (*Oryza* spp.) provides a unique model for studying adaptive evolution because herbicide resistance can arise through both crop‐to‐weed gene flow and independent mutational events. In Brazil, nearly two decades of cultivation of imidazolinone (IMI)‐resistant rice have imposed intense selection for acetolactate synthase (ALS) inhibitors resistance. This study investigated how historical gene flow and independent evolution have shaped the contemporary landscape of ALS resistance mutations in Brazilian weedy rice. Specifically, we characterized the diversity and distribution of ALS resistance mutations and developed rapid allele‐specific molecular markers for major resistance alleles. A total of 200 weedy rice accessions collected from the major rice‐growing region of Brazil were screened for IMI resistance and genotyped using newly developed SNAP (single nucleotide amplified polymorphism) qPCR markers targeting the IMI rice‐derived mutations Ala122Thr, Ser653Asn, and Gly654Glu. Samples negative for these mutations were sequenced, and dose‐response assays were conducted with imazethapyr, penoxsulam, nicosulfuron, and cloransulam‐methyl. Ninety‐three percent of the accessions were resistant to IMI herbicides. The ALS mutation Gly654Glu, present in rice cultivars used until 2006, was found in 15.5% of weedy rice resistant accessions, Ala122Thr became the dominant mutation (54.5%), whereas Ser653Asn occurred at an intermediate frequency (16.0%), indicating a temporal shift in the genetic basis of ALS resistance. Sequencing further identified the ALS substitutions Ala205Val, Trp574Leu, and Ser653Thr, which were not associated with commercial IMI‐resistant rice cultivars, indicating ongoing independent evolution of target‐site resistance. Trp574Leu conferred broad cross‐resistance to all ALS‐inhibiting herbicides evaluated, whereas the remaining mutations displayed mutation‐specific resistance spectra. The SNAP markers showed high specificity and sensitivity, enabling reliable high‐throughput detection of resistance alleles. Together, our findings demonstrate that the contemporary ALS resistance landscape in Brazilian weedy rice has been shaped mainly by repeated crop‐to‐weed gene flow, but also ongoing independent mutational events that continue to diversify resistance under herbicide selection.

## 1 Introduction

Weedy rice (*Oryza* spp.) is one of the most important weed species in rice crop systems worldwide (Clements et al. 2025). Because it belongs to the same species as cultivated rice ( *Oryza sativa* L.), weedy rice shares several morphophysiological traits with the crop, which severely limits the efficacy of available control strategies. The management of weedy rice in rice production systems relies primarily on chemical control. Acetolactate synthase (ALS)‐inhibiting herbicides gained major importance for weedy rice control following the introduction of the Clearfield system, which is based on rice cultivars resistant to imidazolinone (IMI) herbicides, hereafter IMI‐resistant rice (Sudianto et al. 2013). The availability of IMI‐resistant rice in the early 2000s in the USA, South America and Europe enabled the selective control of weedy rice and other important weed species, such as *Echinochloa* spp., *Cyperus* spp., and broadleaf weeds, which indirectly allowed higher crop yield and greater economic return on investment (Avila et al. 2021; Sudianto et al. 2013). However, ALS resistant weedy rice populations were identified only three to four years after the adoption of IMI‐resistant rice (Merotto et al. 2016).

IMI‐resistant rice cultivars carry mutations in the *ALS* gene resulting in amino acid substitutions Gly654Glu (first generation), and Ala122Thr and Ser653Asn (second generation) of the ALS enzyme (Avila et al. 2021). Resistant weedy rice populations carrying the same target‐site resistance mutations as the cultivated rice were found in many regions where IMI‐resistant rice was adopted (Busconi et al. 2012; Presotto et al. 2024; Roso et al. 2010; Singh et al. 2017; Unan et al. 2024). In most of the cases, pollen‐mediated gene flow from IMI‐resistant rice to weedy rice was confirmed as the source of resistance, even though outcrossing rates between these predominantly self‐pollinating species are lower than 1% in the vast majority of cases (Goulart et al. 2016; Shivrain et al. 2009). Although gene flow from IMI‐resistant cultivars is the predominant source of resistance to weedy rice, independent evolution of resistance has also been reported, occurring at a low frequency of 1.1% (Goulart, Matzenbacher, and Merotto 2012; Goulart, Pacheco, et al. 2012). This finding indicates that new mutations may arise and result in resistance similarly to the mutations present in the IMI‐resistant cultivars. Currently, substitutions causing ALS inhibitors resistance are reported in nine amino acid positions Ala122, Pro197, Ala205, Phe206, Asp376, Arg377, Trp574, Ser653, and Gly654 (Cutti et al. 2026) [positions based on *Arabidopsis thaliana* (L.) Heynh. ALS sequence]. Among them, the substitutions Ala122Thr, Ser653Asn and Gly654Glu are found in commercial IMI‐resistant rice varieties (Avila et al. 2021), whereas Pro197Ser (Fuhua et al. 2021), Ala205Val (Qiu et al. 2020), Ala122Val, Trp574Met, and Trp574Cys (Chen et al. 2021), Trp574Leu and Ser653Ile (Sun et al. 2016), and Ser653Thr (Unan et al. 2024) have been naturally identified or artificially generated in *Oryza* species.

Understanding the mechanisms underlying herbicide resistance and their spatial and temporal distribution is essential for the sustainable management of weedy rice (Clements et al. 2025; Cutti et al. 2026) and for understanding the parallel evolution of gene flow and independent origin of herbicide resistance related mutations (Hendry et al. 2011). Knowledge of resistance mechanisms, particularly target‐site mutations, provides critical insights into the evolutionary pathways of resistance, the potential for cross‐resistance, and the likelihood of resistance spread through gene flow or independent selection events (Comont and Neve 2021). In addition, the availability of rapid diagnostic tools, such as molecular markers, is increasingly important for the early and accurate identification of resistant populations. Molecular diagnostic approaches enable more rapid and efficient detection of herbicide resistance than conventional whole‐plant bioassays, especially in cases where resistance is associated with well‐defined and well‐characterized mutations in target‐site enzymes (Beffa et al. 2012).

Resistance to ALS‐inhibiting herbicides driven by pollen‐mediated gene flow from cultivated rice to weedy rice is a well‐characterized and extensively documented evolutionary process (Burgos et al. 2008; Busconi et al. 2012; Cai et al. 2023; Merotto et al. 2016; Presotto et al. 2024; Singh et al. 2017; Ziska et al. 2015). Beyond pollen‐mediated gene flow, resistance alleles may also emerge from standing genetic variation or evolve through independent de novo mutations (Kreiner et al. 2018). Coupled with the widespread adoption of IMI‐resistant rice cultivars in southern Brazil for more than two decades, these multiple evolutionary pathways make weedy rice a unique model for investigating herbicide resistance adaptation. Furthermore, previous regional surveys of ALS resistance mutations (Roso et al. 2010) provide a valuable historical baseline, enabling direct comparisons of shifts in resistance allele frequencies over approximately two decades. The objective of this study was to investigate the evolutionary processes responsible for the current diversity of ALS resistance alleles in Brazilian weedy rice. Specifically, we tested the hypothesis that the contemporary resistance landscape reflects the combined effects of recurrent crop‐to‐weed gene flow from successive IMI‐resistant rice cultivars and independent de novo evolution of target‐site mutations under continued herbicide selection. We further developed molecular tools to distinguish crop‐derived resistance alleles from independently evolved mutations and evaluated their associated resistance and cross‐resistance phenotypes.

## 2 Material and Methods

### 2.1 Plant Material and Herbicide Resistance Screening

Seeds from 200 individual weedy rice (*Oryza* spp.) plants, hereafter referred to as accessions, were collected from rice fields across Rio Grande do Sul State, Brazil, between the 2018/19 and 2024/25 growing seasons (Table S2). Sampling followed a non‐random, targeted approach, focusing on patches of weedy rice plants that survived herbicide application. The geographic location of each collection site was recorded to characterize the spatial distribution of the sampled accessions. Seeds were stored in paper bags at 4°C until use.

Herbicide resistance screening was conducted under greenhouse conditions at the Federal University of Rio Grande do Sul, Porto Alegre, Brazil (30°4′29.66″S, 51°7′59.94″W). Air temperature was maintained at 25°C ± 5°C under a 14‐h photoperiod supplemented with sodium‐vapor lamps providing a photosynthetic photon flux density between 500 and 600 μmol m^−2^ s^−1^. Approximately 10 seeds from each accession were surface sterilized in 5% sodium hypochlorite for 5 min, rinsed thoroughly with distilled water, and germinated on moist germination paper at 27°C for 5 days. Seedlings approximately 2 cm in length were individually transplanted into 200‐mL plastic pots containing a clay loam soil:potting mix (10:1, v/v) fertilized with 240 kg ha^−1^ of N and 200 kg ha^−1^ of both P_2_O_5_ and K_2_O.

Plants at the three‐ to four‐leaf stage were treated with the commercial mixture imazapyr + imazapic (Kifix, BASF; 525 + 175 g kg^−1^) at 73.5 + 24.5 g a.i. ha^−1^ supplemented with 0.5% (v/v) methylated vegetable oil (Dash, BASF; 375 g L^−1^). Herbicide applications were performed using an automated spray chamber (Generation III, DeVries Manufacturing) equipped with a TP9502EVS nozzle (TeeJet Technologies) calibrated to deliver 200 L ha^−1^. We used three biological replicates, and plants were maintained under continuous flooded conditions throughout the experiment.

Herbicide efficacy was visually assessed 21 days after treatment using a scale ranging from 0 to 100%, where 0 indicated the absence of visible injury and 100 represented complete plant death. Data were subjected to ANOVA and treatment means were grouped using the Scott‐Knott test at *p* ≤ 0.05. Accessions exhibiting less than 80% control were classified as resistant and subsequently selected for molecular characterization.

### 2.2 Development and Validation of SNAP Molecular Markers

To develop molecular markers targeting the ALS resistance alleles present in IMI‐resistant rice cultivars, genomic DNA was extracted from the cultivars IRGA424RI, GURI INTACL, IRGA431CL, SCS121CL, IRGA422CL, and SCS115CL, together with the susceptible cultivar IRGA417. Genomic DNA was isolated following the CTAB protocol (Doyle and Doyle 1987).

The *ALS* coding region encompassing all previously described resistance‐conferring substitutions was amplified using primer pairs previously described (Roso et al. 2010) (Table 1). PCR reactions were prepared for a final concentration of 1× PCR buffer, 2 mM MgCl_2_, 0.2 mM dNTPs, 1 U Platinum Taq DNA polymerase (Invitrogen), 0.4 μM of each primer, 100 ng of genomic DNA, and nuclease‐free water to a final volume of 20 μL. For amplification with primer pair AR6_F/AR6_R, 1× PCR Enhancer (Invitrogen) was included. Amplifications with primer pair AR1_F/AR1_R consisted of an initial denaturation at 95°C for 10 min, followed by 40 cycles of 95°C for 30 s, 55°C for 45 s, and 72°C for 60 s, with a final extension at 72°C for 5 min. Amplification with AR6_F/AR6_R followed a touchdown protocol comprising an initial denaturation at 95°C for 10 min; 15 cycles of 95°C for 45 s, 69°C for 45 s with a decrease of 1°C per cycle, and 72°C for 45 s; followed by 25 cycles at 95°C for 45 s, 50°C for 45 s, and 72°C for 45 s, with a final extension at 72°C for 5 min. PCR products were verified by electrophoresis on 1% agarose gels, purified using ExoSAP‐IT (Applied Biosystems), and sequenced on an ABI 3730 Genetic Analyzer (Applied Biosystems) using the BigDye Terminator v3.1 Cycle Sequencing Kit. Sequence chromatograms were inspected and aligned using SnapGene v6.2.1 (Dotmatics), and polymorphic sites associated with herbicide resistance were used to design allele‐specific single nucleotide amplified polymorphism (SNAP) markers.

**TABLE 1:** Sequence, annealing temperature (Ta), melting temperature (Tm), size of the expected fragment in base pairs (bp), number of cycles required for correct genotyping, and the covered position in the *ALS* gene for the SNAP markers and sequencing primers.

Primer	Sequence (5′‐3′)	Ta (°C)	Cycles	Tm (°C)	Fragment (bp)	Covered position^a^
AR6_F^b^ AR6_R^b^	CCAAGACCGGCCGTAAGAAC TGTCCTTGGGGATGTCGACC	55	40	—	643	Ala122, Ala205
AR1_F^b^ AR1_R^b^	CATTCAGGAGTTGGCATTGA TGGGTCATTCAGGTCAAACA	69/50^c^	40	—	444	Trp574, Ser653, Gly654
T122_F4 A122_R4	TTCGCCTACCCGGGCGTC**A** CGGAAGAGGTGGTTGGTGAT	64	35	86.3	80	Thr122
S653N_F S653_R	GTGCTGCCTATGATCCCAG **A** CTGCCATCACCATCCAGGAT	64	35	80.1	62	Asn653
E654_F S653_R	CTGCCTATGATCCCACGTG**A** CTGCCATCACCATCCAGGAT	64	35	79.5	59	Glu654
ACTINA_F^d^ ACTINA_R^d^	CTCCCCCATGCTATCCTTCG TGAATGAGTAACCACGCTCCG	64	35	81	91	—

*Note:* Bold letter indicates the SNP and underlined letter indicates the SNAP for allele‐specific qPCR.

^a^ Based on *ALS* gene sequence from *Arabidopsis thaliana* .

^b^ (Roso et al. 2010).

^c^ Touchdown protocol.

^d^ (Caldana et al. 2007).

Three SNAP primer pairs (Table 1) were designed to specifically detect the ALS substitutions Ala122Thr, Ser653Asn, and Gly654Glu. Primer design followed the SNAP strategy, in which the 3′ terminal nucleotide of the forward primer corresponded to the target SNP, and an additional artificial mismatch was introduced within the four terminal nucleotides to maximize allele specificity. Amplicons were designed to be shorter than 100 bp to enable detection by qPCR. Cycling conditions were subsequently optimized to maximize amplification specificity.

Marker specificity was validated using genomic DNA from cultivars carrying each target mutation as IRGA424RI and GURI INTACL (Ala122Thr), IRGA431CL and SCS121CL (Ser653Asn), IRGA422CL and SCS115CL (Gly654Glu), together with the susceptible cultivar IRGA417 (wild type). Four technical replicates were analyzed for each genotype, and no‐template controls (NTC) were included in every assay. qPCR reactions were performed in a final volume of 10 μL containing 4.6 μL PowerUp SYBR Green Master Mix (Applied Biosystems), 50 ng genomic DNA, 0.2 μM of each primer, and nuclease‐free water. Amplifications were carried out in a QuantStudio 3 Real‐Time PCR System (Applied Biosystems) using the following program: 50°C for 2 min, 95°C for 2 min, followed by 35 cycles of 95°C for 15 s and 64°C for 60 s. Melt‐curve analysis consisted of 90°C for 15 s, 60°C for 60 s, and a gradual increase to 90°C at 0.1°C s^−1^. Fluorescence data were analyzed using Design and Analysis Software v2.6 (Thermo Fisher Scientific), with the fluorescence threshold automatically normalized (ΔRn = 0.04) and melt curves evaluated using a single‐peak detection threshold corresponding to 10% of the dominant peak.

Primer efficiency was further evaluated using 10‐fold serial dilutions of genomic DNA obtained from cultivars carrying each target mutation and the susceptible control. Standard curves generated from four technical replicates were used to estimate amplification efficiency according to *E* = 100 (10^(−1/slope)−1).

To evaluate marker sensitivity for high‐throughput molecular screening, composite leaf samples were assembled using 1 cm diameter leaf discs collected from resistant and susceptible plants in resistant:susceptible proportions ranging from 1:0 to 1:50. Composite DNA samples were amplified using the corresponding SNAP marker together with the primer for endogenous reference gene *actin1* (Os03g0718100). Normalized Cq values (Cq_target ‐ Cq_actin1) were plotted against the proportion of resistant plants to determine the minimum detectable frequency of each resistance allele. Marker applicability was subsequently assessed using independent composite samples containing resistant:susceptible proportions ranging from 1:10 to 10:0. This approach aimed to estimate, based on the composite leaf discs, the proportion of plants carrying each ALS resistance mutation within the sample. Two biological replicates and six technical replicates per target were analyzed, and normalized Cq values were fitted to linear regression models to estimate the proportion of resistant individuals within composite samples.

### 2.3 Weedy Rice Genotyping

The 200 weedy rice accessions screened for resistance to IMI herbicides were subsequently genotyped to determine the ALS resistance mutations underlying the resistant phenotype. Genomic DNA was extracted from approximately 0.1 g of leaf tissue collected from one representative plant per accession following the protocol described above. DNA quality was initially verified by amplifying the housekeeping *actin1* gene (Table 1), and samples showing successful amplification were subjected to mutation‐specific genotyping.

All accessions were first screened using the SNAP molecular markers developed for the three ALS substitutions from IMI‐resistant rice cultivars, namely Ala122Thr, Ser653Asn, and Gly654Glu. qPCR reactions were performed under the conditions described in Section 2.2 using two technical replicates per biological sample. The rice cultivars IRGA424RI, IRGA431CL, and IRGA422CL served as positive controls for Ala122Thr, Ser653Asn, and Gly654Glu, respectively, whereas two NTC were included in each qPCR plate to monitor reagent contamination. Amplification specificity was confirmed by melt‐curve analysis. Samples were considered positive when they exhibited successful amplification together with a single melting peak corresponding to the expected amplicon.

Resistant accessions that failed to amplify with any of the three SNAP markers were subjected to Sanger sequencing of the ALS gene using the primer pairs described in Section 2.2 to identify alternative mutations. Sequence analysis revealed three additional ALS substitutions that were not derived from IMI‐resistant rice cultivars, namely Ala205Val, Trp574Leu, and Ser653Thr. Based on these polymorphisms, an additional set of allele‐specific SNAP markers was designed following the same criteria described for the original markers (Figure S1 and Table S3). The complete collection of weedy rice accessions was subsequently re‐genotyped using these newly developed markers to determine the distribution and frequency of all identified ALS resistance alleles.

### 2.4 Characterization of ALS Resistance Mutations

The new ALS substitutions identified during the genotyping (Ala205Val, Trp574Leu, and Ser653Thr) had not previously been associated with the IMI‐resistant rice cultivars grown in Brazil. To characterize their resistance profiles and compare them with the mutations introgressed from cultivated rice (Ala122Thr, Ser653Asn, and Gly654Glu), representative weedy rice accessions carrying each mutation were selected for herbicide dose‐response experiments. The susceptible rice cultivar IRGA417 was included as a reference.

Four ALS‐inhibiting herbicides representing different chemical families were evaluated: imazethapyr (Imazetapir Plus Nortox, Nortox; 200 g L^−1^, SL) applied at rates ranging from 0 to 3200 g a.i. ha^−1^ with 0.5% (v/v) mineral oil (Assist EC, BASF; 782 g L^−1^); nicosulfuron (Nicosulfuron Nortox 40 SC, Nortox; 40 g L^−1^, SC) applied at rates ranging from 0 to 240 g a.i ha^−1^ with 0.1% (v/v) mineral oil; cloransulam‐methyl (Pacto, Corteva; 840 g kg^−1^, WG) applied at rates ranging from 0 to 120 g a.i. ha^−1^ with 0.2% (v/v) nonionic surfactant based on alkylphenol ethoxylates (Haiten, UPL; 200 g L^−1^); and penoxsulam (Ricer, Corteva; 240 g a.i. L^−1^, SC) applied at rates ranging from 0 to 1920 g a.i. ha^−1^ with 0.5% (v/v) vegetable oil fatty acid esters (Veget'oil, Oxiquímica; 930 g L^−1^).

Plants were established and maintained under the same greenhouse conditions described for the resistance screening (Section 2.1). Herbicides were applied at the three‐ to four‐leaf stage using the same automated spray chamber calibrated to deliver 200 L ha^−1^. Each treatment included four biological replicates, and the entire experiment was conducted twice.

Fresh aboveground biomass was determined 21 days after treatment. Data from the two experimental runs were initially evaluated using Levene's test to assess homogeneity of variance. As no significant differences between runs were detected (*p* ≤ 0.05), datasets were pooled (*n* = 8) before statistical analysis. Dose‐responses were analysed in R using the *drc* package by fitting a three‐parameter log‐logistic model: *y* = *d*/[1 + (*x*/*e*) ^ *b*], where *y* represents fresh biomass, *x* is the herbicide dose, *d* is the upper asymptote, *b* is the slope around the inflection point, and *e* corresponds to the herbicide rate causing a 50% reduction in biomass (GR_50_). Resistance indices (RI) were calculated as the ratio between the GR_50_ of each resistant accession and that of the susceptible cultivar IRGA417. Dose‐response curves were generated using *ggplot2*.

## 3 Results

### 3.1 SNAP Molecular Marker Sensitivity and High‐Throughput Utilization

The SNAP molecular markers with optimized reactions were highly specific to the mutations Ala122Thr, Ser653Asn, and Gly654Glu (Figure 1). No primer produced amplification for the wild‐type genotype before the 35th cycle during the validation, and all SNAP markers successfully identified their target mutations in two rice cultivars. The efficiency of the allele‐specific primers was 135.7%, 121.4%, and 113.0% for the markers for Ala122Thr, Ser653Asn, and Gly654Glu, respectively (Suppl. Fig. 2).

**FIGURE 1:**
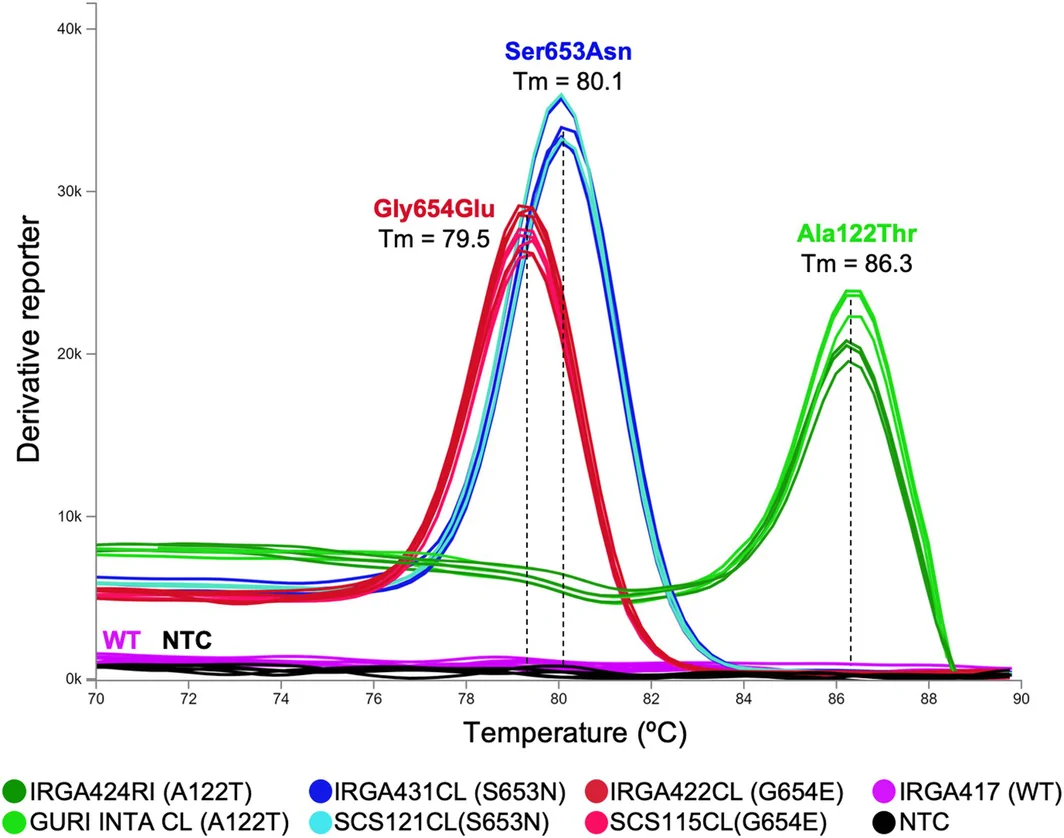
Melt curve plot for rice cultivars and the three SNAP markers related to the ALS mutations from IMI‐resistant rice Ala122Thr (A122T), Ser653Asn (S653N), and Gly654Glu (G654E).

The SNAP markers for Ala122Thr and Ser653Asn successfully identified one resistant plant in a sample of 50, while the marker for Gly654Glu detected one resistant plant among 40 (Figure S3). When the sample size was reduced to 10 plants, normalized Cq decreased with increasing numbers of resistant plants, with the strongest linear relationship observed for Ala122Thr (*R* ^2^ = 0.98). For Ser653Asn and Gly654Glu, the Cq decreased markedly as the number of resistant plants increased up to approximately five plants per sample, after which Cq values tended to stabilize. Accordingly, the relationships were weaker for Ser653Asn (*R* ^2^ = 0.82) and Gly654Glu (*R* ^2^ = 0.78) (Figure S3).

### 3.2 Frequency and Distribution of ALS Resistance Mutations

Of the 200 weedy rice accessions evaluated, 186 were classified as resistant to IMI herbicides. Among these, 109, 32, and 31 carried the crop‐derived IMI‐resistance mutations Ala122Thr, Ser653Asn, and Gly654Glu, respectively (Figure 2). Three accessions carried both Ala122Thr and Gly654Glu mutations (Figure 3). Eleven accessions were resistant due to mutations not associated with IMI‐resistant rice cultivars. Of these, nine carried Ala205Val, one carried Trp574Leu, and one carried Ser653Thr (Figure 3 and Figure S4). No clear spatial pattern was detected in the distribution of ALS resistance mutations across the sampled regions (Figure 2). The western border region showed a higher predominance of Ala122Thr, although samples from this region represented less than 15% of the total accessions collected.

**FIGURE 2:**
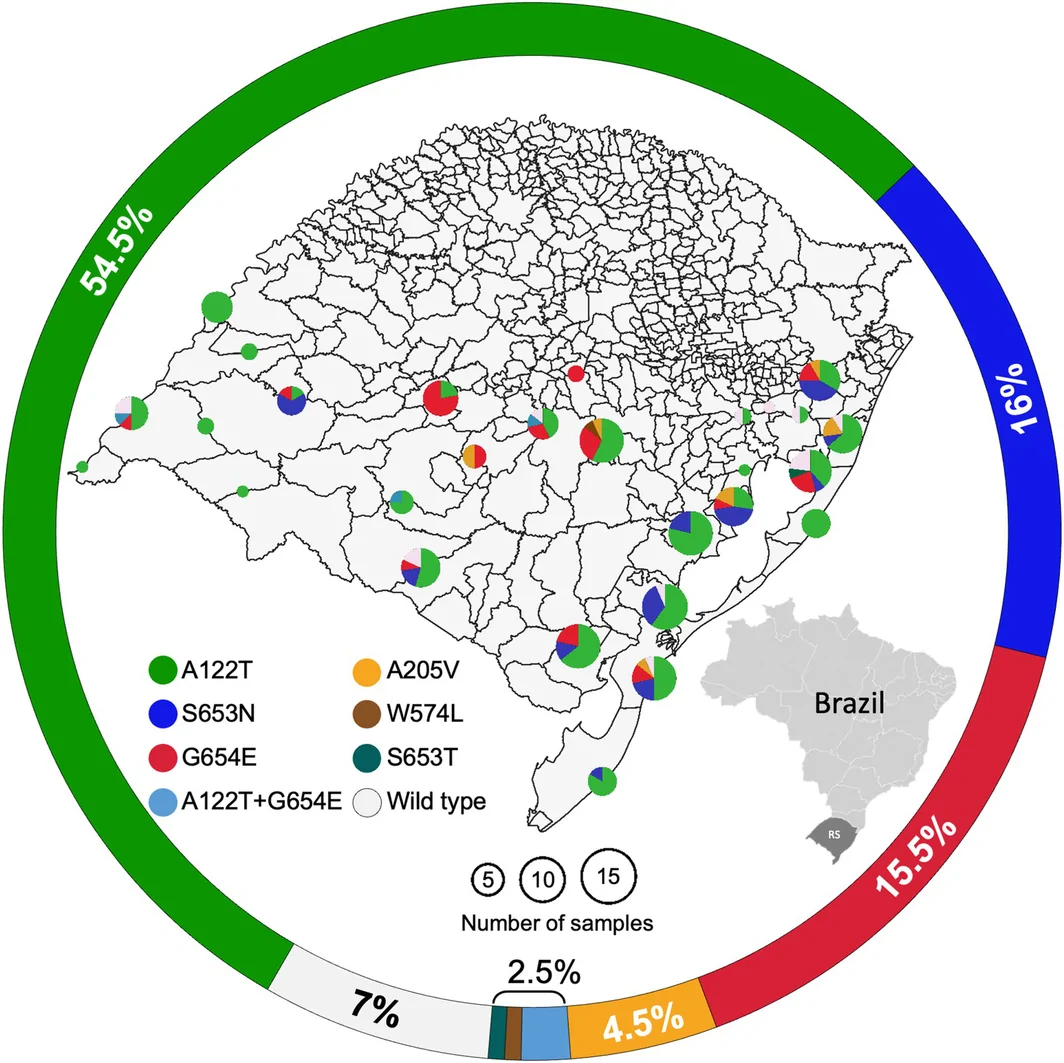
Frequency and geographic distribution of acetolactate synthase (ALS) resistance mutations in weedy rice populations from Rio Grande do Sul, Brazil. Percentages displayed in the outer ring represent the proportion of each mutation. Pie charts indicate the relative frequency of ALS genotypes detected within each sampled population, with colors representing the mutations Ala122Thr (A122T), Ser653Asn (S653N), Gly654Glu (G654E), Ala205Val (A205V), Trp574Leu (W574L), Ser653Thr (S653T), the double mutant A122T + G654E, and wild‐type individuals. Pie chart size is proportional to the number of individuals analyzed per population.

**FIGURE 3:**
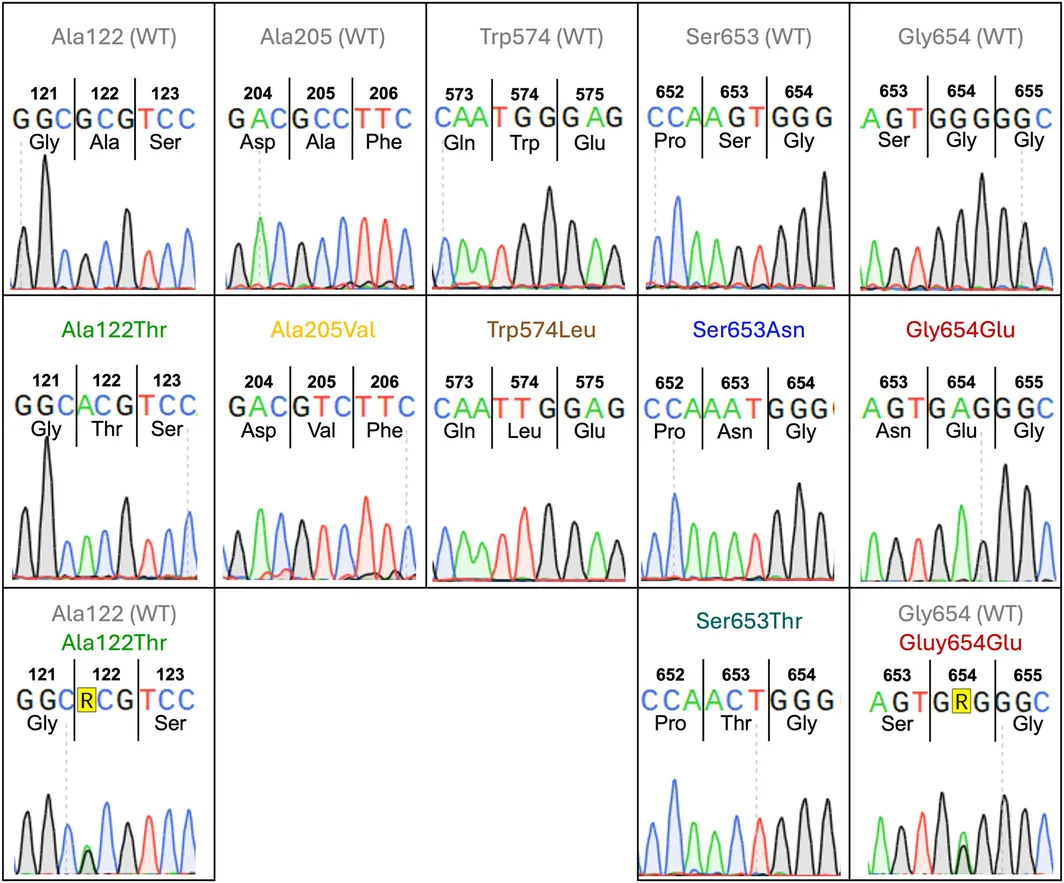
Sequencing chromatograms of representative weedy rice accessions of the wild type (WT) or carrying the Ala122Thr, Ala205Val, Trp574Leu, Ser653Asn, Ser653Thr, or Gly654Glu mutations. Double peaks indicate heterozygosity. Nucleotide codes: A, adenine; C, cytosine; G, guanine; R, adenine or guanine; T, thymine.

### 3.3 Functional Characterization of ALS Resistance Mutations

The dose‐response analyses revealed pronounced differences in herbicide sensitivity among weedy rice genotypes carrying different ALS mutations (Figure 4 and Table S1). Herbicides representing four ALS‐inhibiting chemical families—imidazolinones (IMI), sulfonylureas (SU), triazolopyrimidine type 1 (TPD‐1), and triazolopyrimidine type 2 (TPD‐2) were used to evaluate the cross‐resistance pattern conferred by individual ALS mutations. The Trp574Leu substitution conferred the broadest resistance spectrum, with the carrier accession remaining unaffected across all herbicides and doses evaluated (Figure 4 and Table S1). The Ala122Thr mutation conferred the highest level of resistance to IMIs (RI = 139.5), whereas Ser653Asn (RI = 86.5), Ala205Val (RI = 78.2), and Ser653Thr (RI = 61.0) conferred medium‐level resistance. Gly654Glu conferred low‐level resistance (RI = 10.6) (Table S1).

**FIGURE 4:**
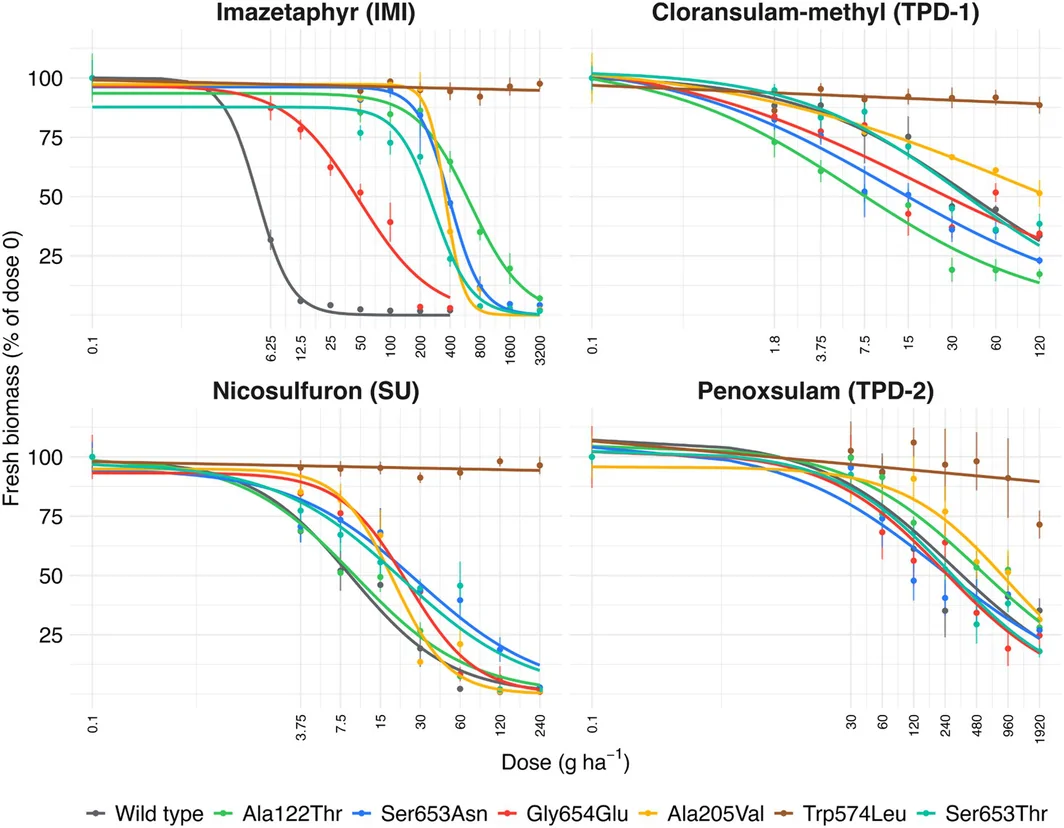
Dose–response curves for fresh biomass (% of the untreated control) of plants treated with imazethapyr (IMI, imidazolinone), cloransulam‐methyl (TPD‐1, triazolopyrimidine‐1), nicosulfuron (SU, sulfonylurea), and penoxsulam (TPD‐2, triazolopyrimidine‐2). Curves represent different ALS genotypes, including wild‐type plants and those carrying the Ala122Thr, Ser653Asn, Gly654Glu, Ala205Val, Trp574Leu, and Ser653Thr substitutions. Points indicate mean (*n* = 8) values, lines show the fitted dose–response models, and bars represent the standard error of the mean.

Penoxsulam (TPD‐2) showed limited differentiation among genotypes. A gradual reduction in biomass occurred across increasing doses, including in the wild‐type accession (Figure 4). For nicosulfuron, the wild‐type and the Ala122Thr accession exhibited a GR_50_ of approximately 15% of the label rate (60 g ha^−1^) (Table S1). In contrast, the accessions carrying Ser653Asn, Gly654Glu, Ala205Val, and Ser653Thr showed greater tolerance to increasing nicosulfuron rates, with RI values ranging from 2.1 to 3.1 (Table S1). Cloransulam‐methyl produced GR_50_ values generally similar to or lower than that of the wild‐type, resulting in RI values below to 1.0 for most genotypes (Table S1).

## 4 Discussion

### 4.1 Rapid Detection of Herbicide Resistance Mutations

The SNAP molecular markers provided a fast and precise method to screen for herbicide resistance mutations. Allele‐specific assays based on end‐point PCR (Kadaru et al. 2008; Roso et al. 2010), dCAPS (Li et al. 2025), and KASP (Rosas et al. 2014) have already been developed for known mutations. However, the first two approaches may be limited in terms of effectiveness and applicability, whereas the latter may involve relatively high reagent costs. Therefore, we developed a set of allele‐specific primers for high‐throughput and low‐cost SNP genotyping related to imidazoline herbicide resistance in *Oryza* spp. The observed efficiency values over 100% may be related to inhibitory effects in more concentrated samples which can lead to a deviation in the standard curve (Svec et al. 2015). Despite being highly specific, the presence of a big amount of wild‐type DNA may jeopardize primer efficiency (Zhao et al. 2021). Moreover, DNA quantity may vary in function of sampling and extraction protocols. Therefore, we recommend no more than 20 individual samples per composite sample to ensure the correct diagnosis of mutations present in the area. From a practical standpoint, when the required outcome is limited to the presence or absence of resistance within a field, the use of normalized Ct values is not necessary. Under these conditions, analyzing up to 20 individual samples per composite sample provides an adequate margin of error to account for potential sampling bias or failures.

The correlation between number of resistant plants and normalized Cq for the marker for Ala122Thr (Figure S3) can be used to estimate the number of resistant plants in a composite sample of up to 10 plants, considering the same amount of tissue collected for each plant. In the same conditions, data for the markers Ser653Asn and Gly654Glu may be used to detect up to five resistant plants but cannot differentiate samples containing from 6 to 10 resistant plants (Figure S3). It is recommended that these calibration curves be recalibrated using the highest possible number of replicates, considering locally available reagents and protocols, to ensure their most accurate application. This approach is valuable because, at the field scale, it is common to find plants carrying different resistance mutations, and this method allows the rapid estimation of the frequency of each mutation within a field.

### 4.2 Evolutionary Dynamics of ALS Resistance Alleles

The high frequency of the Ala122Thr mutation is consistent with the extensive use of IMI‐resistant rice cultivars carrying this mutation, such as IRGA424RI, GURI INTACL, and PUITA INTACL (Avila et al. 2021). These cultivars have accounted for at least 80% of the rice‐seeded area in southern Brazil over the past decade (IRGA 2021). In contrast, we observed a relatively high frequency of the Gly654Glu mutation (Figure 2). Despite no longer being deployed in commercial cultivars for more than 15 years, the Gly654Glu allele persisted in contemporary weedy rice populations. The maintenance of this first‐generation resistance allele suggests that resistance alleles introduced through crop‐to‐weed introgression may remain in populations long after the original selective source has disappeared. Such persistence is likely promoted by a combination of seed‐bank recruitment (Andres et al. 2021), recurrent gene flow among resistant weedy rice populations, and potentially limited fitness penalties associated with these mutations (Goulart, Matzenbacher, and Merotto 2012; Goulart, Pacheco, et al. 2012).

The current frequency of ALS resistance alleles reveals that the genetic architecture of herbicide resistance in Brazilian weedy rice has changed substantially since the introduction of the Clearfield production system. Previous studies based on SNAP molecular markers carried out during 2006/2007 and 2007/2008 growing seasons identified frequencies of 95.4% of Gly654Glu, followed by 26.7% of Ser653Asn and 10.8% of Ala122Thr mutations (Roso et al. 2010). This turnover in the predominant resistance allele closely mirrors the replacement of first‐generation IMI‐resistant rice cultivars by second‐generation cultivars carrying the Ala122Thr and Ser653Asn mutations (Avila et al. 2021; IRGA 2021), highlighting the long‐term impact of repeated crop‐to‐weed gene flow on the evolutionary dynamics of herbicide resistance. In accordance with our data, a global‐scale study reported that, out of 60 IMI‐resistant weedy rice genotypes, 44%, 16%, and 40% carried the Ala122Thr, Ser653Asn, and Gly654Glu mutations, respectively (Qiu et al. 2020). In our study, only three accessions carried both Ala122Thr and Gly654Glu mutations. In contrast with our findings, a previous study (Roso et al. 2010) reported a high frequency of plants carrying two mutations. This inconsistency may be partly explained by limitations of the end‐point PCR method and non–specificity of the allele‐specific primers used at the time. Nevertheless, the occurrence of multiple alleles of resistance within the same individual provides evidence that gene flow between resistant genotypes is still occurring. However, the low frequency of double mutants and their exclusive occurrence in the heterozygous state (Figure 3) may indicate selective constraints against multiple target‐site mutations within the same *ALS* locus, possibly reflecting fitness costs associated with this genetic architecture (Vila‐Aiub 2019).

We identified weedy rice accessions carrying ALS substitutions not associated with IMI‐resistant rice cultivars, including Ala205Val, Trp574Leu, and Ser653Thr. The identification of this novel mutation demonstrates that ALS resistance evolution in Brazilian weedy rice is not driven exclusively by introgression from cultivated rice. Instead, these substitutions indicate that independent mutational events are contributing to the diversification of resistance alleles under continued herbicide selection. Similarly, previous studies have shown that independently evolved ALS resistance mutations occur at low frequencies, typically representing less than 5% of resistant populations (Unan et al. 2024). The Ala205Val mutation has previously been reported at low frequencies in Brazilian weedy rice populations (Qiu et al. 2020), whereas Ser653Thr has been identified as a naturally occurring resistance allele in weedy rice populations from the United States (Unan et al. 2024). In contrast, to our knowledge, this is the first report of Trp574Leu occurring naturally in weedy rice populations. The Trp574Leu substitution is one of the most widespread ALS resistance mutations across weed species and is well known to confer broad cross‐resistance to all chemical families of ALS‐inhibiting herbicides (Cutti et al. 2026). Although this mutation has previously been introduced into cultivated rice through genome editing (Sun et al. 2016), its evolutionary fate following its emergence in natural weedy rice populations remains unknown.

The coexistence of crop‐derived and independently evolved resistance mutations suggests that both gene flow and independent mutation contribute to the current ALS resistance landscape in weedy rice. Although pollen‐mediated gene flow is a well‐established mechanism driving the evolution of weedy rice (Wedger et al. 2022), it is still unclear how much the spread of the Ala122Thr, Ser653Asn, and Gly654Glu alleles resulted from crop‐to‐weed versus weed‐to‐weed gene flow. Previous studies have shown that independent origins of ALS resistance mutations are common (Kreiner et al. 2022) and that the same mutations found in IMI‐resistant rice cultivars can also arise independently through standing genetic variation or de novo mutation, although at low frequencies (Wedger et al. 2022). Therefore, while the high frequency of Ala122Thr, Ser653Asn, and Gly654Glu strongly suggests that gene flow has been the main source of these resistance alleles, the available data do not allow us to determine the exact origin of each resistant accession. Likewise, we cannot determine whether the nine accessions carrying the Ala205Val mutation originated from a single independent mutation followed by seed dispersal or by gene flow among weedy rice populations or from multiple independent mutation events.

The absence of a clear spatial distribution may be attributed to an imbalance in sampling the seeds. The largest number of samples was collected in the inner and outer coastal plain regions of Rio Grande do Sul, and the mutation frequencies observed within each sampling site largely reflected the overall mutation frequency patterns identified in the entire dataset. The apparent enrichment of Ala122Thr mutation from the western border region should be interpreted with caution, as it may reflect sampling intensity rather than a true regional differentiation in mutation prevalence. The absence of a clear geographic structure is consistent with previous studies showing that the regional spread of IMI‐resistant weedy rice in southern Brazil has been driven primarily by long‐distance seed dispersal, rather than by successive pollen‐mediated gene flow events (Merotto et al. 2016).

### 4.3 Functional Characterization of ALS Resistance Mutations and Agronomic Implications

IMI herbicides are extensively used for weedy rice control (Avila et al. 2021) and therefore represent the primary selection pressure driving the evolution of ALS resistance. Variation in the resistance level conferred by different ALS mutations could partly explain their frequencies in the population. However, the frequency of these mutations (Figure 2) did not correspond to the level of resistance they conferred (Figure 4). For example, Gly654Glu was among the most frequent resistance alleles despite conferring only low‐level IMI resistance, whereas Trp574Leu, which conferred the broadest resistance spectrum, was detected in only a single accession. These findings indicate that the evolutionary success of ALS resistance alleles cannot be explained solely by resistance level but is mainly influenced by factors such as repeated introgression from IMI‐resistant rice cultivars and the strength and duration of herbicide selection, as observed in another study (Kreiner et al. 2018).

The TPD‐2 herbicide penoxsulam is considered a safe herbicide for use in rice production systems, with selectivity allowing application both at pre‐ and post‐emergence stages (Bond et al. 2007). Consequently, the resistance mutations identified in this study are unlikely to be strongly selected by penoxsulam use alone, suggesting that this herbicide is unlikely to substantially alter the current frequency and distribution of ALS resistance alleles in the short term. The SU and TPD‐1 herbicides used in our study are not registered or routinely used in rice. Consequently, resistance responses to these herbicides reflect the intrinsic cross‐resistance spectrum associated with each ALS mutation rather than adaptation driven by direct selection in rice production systems. If future herbicide‐resistant rice technologies enable the use of these herbicides, the resistance profiles observed here (Figure 4) suggest that they may impose relatively weak selection on most ALS resistance alleles, like penoxsulam. Under this scenario, they would be expected to have a limited influence on short‐term changes in allele frequencies, although this hypothesis should be validated under field conditions.

The Trp574Leu mutation was detected at low frequency but conferred a high level of herbicide resistance (Figure 4). This mutation represents a highly adaptive allele under strong ALS inhibitor selection due to its ability to confer resistance across multiple chemical groups (Yu et al. 2010). In contrast, the predominant mutations Ala122Thr, Ser653Asn, and Gly654Glu, which were largely introduced through gene flow from IMI‐resistant rice cultivars, exhibited lower resistance levels, suggesting differences in the adaptive value of individual ALS alleles under long‐term herbicide selection. Currently, despite the large occurrence of weedy rice resistant to ALS inhibitors, farmers still use IMI‐resistant rice cultivars, often accompanied by repeated or increased herbicide application ratios.

From an agronomic perspective, herbicide choice should account for the specific ALS resistance allele present in the population rather than assuming uniform cross‐resistance across all ALS inhibitors (Cutti et al. 2026; Rudell et al. 2025). In contrast, our findings indicate that, in weedy rice, differences in the level of resistance conferred by individual ALS alleles may have limited practical relevance for herbicide choice, as variation in resistance levels among IMI herbicides did not appear to substantially affect the frequencies of the corresponding resistance alleles in the populations. The low frequency of broad‐spectrum resistance mutations such as Trp574Leu highlights an opportunity to delay their spread through diversified weed management, including herbicide rotation, integration of nonchemical practices, and reduction of repeated selection imposed by IMI‐resistant rice systems. In addition, sulfonylureas could become a viable option if future changes in rice cultivars allow its selective use. The availability of rapid SNAP markers further enables early detection of resistance alleles, supporting more informed herbicide recommendations and regional resistance monitoring programs. A future observation would be the frequency of the high‐level resistant mutation Trp574Leu over the actual predominant mutations associated with gene flow from rice cultivars, and in interaction with the new developed weedy rice control system based on resistance to Acetyl‐CoA carboxylase (ACCase)‐inhibiting herbicides (Chiapinotto et al. 2026; Chiapinotto et al. 2024; Turra et al. 2026).

## 5 Conclusions

This study demonstrates that ALS resistance in Brazilian weedy rice is widespread, genetically diverse, and shaped by the combined effects of historical crop‐to‐weed gene flow and independent mutational events. Resistance alleles introgressed from IMI‐resistant rice cultivars remain predominant in Brazilian weedy rice populations. Ala122Thr has replaced Gly654Glu as the predominant ALS resistance mutation, indicating a temporal shift in the genetic basis of resistance. Despite this shift, the first‐generation Gly654Glu allele remains present in contemporary populations. Furthermore, the identification of additional independently evolved ALS substitutions demonstrates that the genetic architecture of resistance continues to diversify under repeated herbicide selection. The allele‐specific SNAP molecular markers developed here provide a rapid, cost‐effective, and scalable diagnostic approach that can be readily applied for early detection and monitoring of resistance alleles at the field and regional levels, and for future studies related to the evolution of ALS resistance in weedy rice. Together, these findings contribute critical insights into the evolutionary dynamics of ALS resistance in weedy rice and support the development of more robust, mutation‐informed weed management strategies aimed at preserving herbicide efficacy and sustaining rice production systems.

## Funding

Funding was provided by the National Council for Scientific and Technological Development (CNPq) under the project 404500/2023‐4, and the Rio Grande do Sul State Research Support Foundation (FAPERGS), Brazil, Grant No. 22/2551‐0000394‐0.

## Conflicts of Interest

The authors declare that the molecular markers and plant materials carrying the ALS resistance mutations described in this manuscript are covered by patent application BR1020260149047. G.M.T., P.S.A., and A.M.J. are named as inventors on this application.

## Supporting information


**Figure S1:** Validation of SNAP molecular markers for mutations not related to imidazoline‐resistant rice cultivars. Melt curve plots with single peaks for weedy rice accessions and the three SNAP primers pairs for the new ALS substitutions. IRGA417 was used as negative control and NTC is the non‐template control. Tm: melt temperature.
**Figure S2:** Standard curves used to estimate the SNAP molecular markers efficiency (%) developed for the detection of ALS resistance mutations in weedy rice (A) Ala122Thr (A122T), (B) Ser653Asn (S653N), and (C) Gly654Glu (G654E).
**Figure S3:** Sensitivity and calibration of SNAP molecular markers targeting the ALS resistance mutations Ala122Thr (A–D), Ser653Asn (B–E), and Gly654Glu (C–F). For sensitivity analysis (A–C), resistant and susceptible DNA samples were mixed at defined proportions (1:50, 1:40, 1:30, 1:20, 1:10, and 1:0), and marker performance was evaluated using normalized quantification cycle (ΔCq) values relative to the actin1 reference gene. Values represent the mean ± standard deviation of four technical replicates. Calibration curves (D–F) show the relationship between normalized ΔCq values and the number of resistant plants in composite samples containing up to 10 individuals.
**Figure S4:** Alignment of partial sequences of the *Oryza sativa* ALS gene with the reference sequence DQ516980.1, generated from sequencing of the two regions of the ALS gene (primers AR6 and AR6, Table 1). Nucleotides highlighted in yellow represent nonsynonymous mutations.
**Table S1:** Parameter estimates of the three‐parameter log‐logistic model (LL.3) describing the dose–response relationship for fresh biomass of weedy rice accessions carrying different mutations treated with imazethapyr, penoxsulam, nicosulfuron, and cloransulam‐methyl.
**Table S2:** Weedy rice accessions information and control efficacy (%) after imazapyr + imazapic treatment (*n* = 3).
**Table S3:** Sequence, annealing temperature (Ta), melting temperature (Tm), size of the expected fragment in base pairs (bp), number of cycles required for correct genotyping, and the covered position in the ALS gene for the SNAP markers and sequencing primers.

## Data Availability

Supporting Information is available in the online version of this manuscript. The data supporting the findings of this study are available from the corresponding author upon reasonable request.

## References

[eva70342-bib-0001] Andres, A. , S. Fogliatto , L. Bastiaans , and F. Vidotto . 2021. “Dynamics of Weedy Rice Soil Seedbank Under Different Control Strategies in Italian Rice Fields: Survey and Model Study.” Weed Science 69: 575–584. 10.1017/wsc.2021.51.

[eva70342-bib-0014] Avila, L. A. , E. Marchesan , E. R. Camargo , et al. 2021. “Eighteen Years of Clearfield Rice in Brazil: What Have We Learned?” Weed Science 69: 585–597. 10.1017/wsc.2021.49.

[eva70342-bib-0002] Beffa, R. , A. Figge , L. Lorentz , et al. 2012. “Weed Resistance Diagnostic Technologies to Detect Herbicide Resistance in Cerealgrowing Areas. A Review.” Julius‐Kühn‐Archiv 434, no. 1: 75–80. 10.5073/JKA.2012.434.008.

[eva70342-bib-0003] Bond, J. A. , T. W. Walker , E. P. Webster , N. W. Buehring , and D. L. Harrell . 2007. “Rice Cultivar Response to Penoxsulam.” Weed Technology 21: 961–965. 10.1614/WT-07-003.1.

[eva70342-bib-0004] Burgos, N. R. , J. K. Norsworthy , R. C. Scott , and K. L. Smith . 2008. “Red Rice ( *Oryza sativa* ) Status After 5 Years of Imidazolinone‐Resistant Rice Technology in Arkansas.” Weed Technology 22: 200–208. 10.1614/WT-07-075.1.

[eva70342-bib-0005] Busconi, M. , D. Rossi , C. Lorenzoni , G. Baldi , and C. Fogher . 2012. “Spread of Herbicide‐Resistant Weedy Rice (Red Rice, *Oryza sativa* L.) After 5 Years of Clearfield Rice Cultivation in Italy.” Plant Biology (Stuttgart, Germany) 14: 751–759. 10.1111/j.1438-8677.2012.00570.x. 22443148

[eva70342-bib-0006] Cai, X.‐X. , Z. Wang , Y. Yuan , L.‐H. Pang , Y. Wang , and B.‐R. Lu . 2023. “Crop–Weed Introgression Plays Critical Roles in Genetic Differentiation and Diversity of Weedy Rice: A Case Study of Human‐Influenced Weed Evolution.” Biology 12: 744. 10.3390/biology12050744. PMC10215757 37237556

[eva70342-bib-0007] Caldana, C. , W.‐R. Scheible , B. Mueller‐Roeber , and S. Ruzicic . 2007. “A Quantitative RT‐PCR Platform for High‐Throughput Expression Profiling of 2500 Rice Transcription Factors.” Plant Methods 3: 7. 10.1186/1746-4811-3-7. PMC1914063 17559651

[eva70342-bib-0008] Chen, L. , G. Gu , C. Wang , et al. 2021. “Trp548Met Mutation of Acetolactate Synthase in Rice Confers Resistance to a Broad Spectrum of ALS‐Inhibiting Herbicides.” Crop Journal 9: 750–758. 10.1016/j.cj.2020.11.003.

[eva70342-bib-0009] Chiapinotto, D. M. , L. A. Avila , D. Agostinetto , et al. 2024. “From the Clearfield to the Provisiatm System for Rice Production: Challenges and Management Opportunities.” Advances in Weed Science 42: e020242042. 10.51694/AdvWeedSci/2024;42:00028.

[eva70342-bib-0010] Chiapinotto, D. M. , L. A. Avila , D. Agostinetto , et al. 2026. “Gene Flow of Quizalofop‐p‐Ethyl Resistance From Crop to Weedy Rice.” Advances in Weed Science 44: e020251107. 10.51694/AdvWeedSci/2026;44:0020.

[eva70342-bib-0011] Clements, D. R. , J. Braithwaite , and L. Mckenna . 2025. “Crops and Weeds Running to Stand Still in the Red Queen's Climate Change Race.” Advances in Weed Science 43: e020250070. 10.51694/AdvWeedSci/2025;43:00015.

[eva70342-bib-0012] Comont, D. , and P. Neve . 2021. “Adopting Epidemiological Approaches for Herbicide Resistance Monitoring and Management.” Weed Research 61: 81–87. 10.1111/wre.12420.

[eva70342-bib-0013] Cutti, L. , E. L. Patterson , T. A. Gaines , and A. Merotto . 2026. “From Knowledge to Action: The Importance of Characterizing Herbicide Resistance Mechanisms for Implementing Management Strategies.” Pest Management Science: ps.71187. 10.1002/ps.71187.42596560

[eva70342-bib-0015] Doyle, J. J. , and J. L. Doyle . 1987. “A Rapid DNA Isolation Procedure for Small Quantities of Fresh Leaf Tissue.” Phytochemical Bulletin 19: 11–15.

[eva70342-bib-0016] Fuhua, W. , L. I. Zichao , W. Ya , et al. 2021. “New Herbicide‐Resistant Japonica Rice Germplasms Created by EMS Mutagenesis.” Journal of Henan Agricultural Sciences 50: 8. 10.15933/j.cnki.1004-3268.2021.04.002.

[eva70342-bib-0017] Goulart, I. C. G. d. R. , M. T. Pacheco , A. L. Nunes , and A. Merotto . 2012. “Identification of Origin and Analysis of Population Structure of Field‐Selected Imidazolinone‐Herbicide Resistant Red Rice ( *Oryza sativa* ).” Euphytica 187: 437–447. 10.1007/s10681-012-0738-5.

[eva70342-bib-0018] Goulart, I. C. G. R. , F. O. Matzenbacher , and A. Merotto . 2012. “Differential Germination Pattern of Rice Cultivars Resistant to Imidazolinone Herbicides Carrying Different Acetolactate Synthase Gene Mutations.” Weed Research 52: 224–232. 10.1111/j.1365-3180.2012.00909.x.

[eva70342-bib-0019] Goulart, I. C. G. R. , V. G. Menezes , E. D. Botoly , V. Kupas , and A. Merotto . 2016. “Detecting Gene Flow From ALS‐Resistant Hybrid and Inbred Rice to Weedy Rice Using Single Plant Pollen Donors.” Experimental Agriculture 52: 237–250. 10.1017/S0014479715000058.

[eva70342-bib-0020] Hendry, A. P. , M. T. Kinnison , M. Heino , et al. 2011. “Evolutionary Principles and Their Practical Application.” Evolutionary Applications 4: 159–183. 10.1111/j.1752-4571.2010.00165.x. PMC3352551 25567966

[eva70342-bib-0021] IRGA . 2021. Cultivares. IRGA. https://irga.rs.gov.br/cultivares.

[eva70342-bib-0022] Kadaru, S. , W. Zhang , A. Yadav , and J. H. Oard . 2008. “Development and Application of Allele‐Specific PCR Assays for Imazethapyr Resistance in Rice ( *Oryza sativa* ).” Euphytica 160: 431–438. 10.1007/s10681-008-9662-0.

[eva70342-bib-0023] Kreiner, J. M. , G. Sandler , A. J. Stern , et al. 2022. “Repeated Origins, Widespread Gene Flow, and Allelic Interactions of Target‐Site Herbicide Resistance Mutations.” eLife 11: e70242. 10.7554/eLife.70242. PMC8798060 35037853

[eva70342-bib-0024] Kreiner, J. M. , J. R. Stinchcombe , and S. I. Wright . 2018. “Population Genomics of Herbicide Resistance: Adaptation via Evolutionary Rescue.” Annual Review of Plant Biology 69: 611–635. 10.1146/annurev-arplant-042817-040038. 29140727

[eva70342-bib-0025] Li, J. , J. Yu , S. Yin , H. Gao , X. Hou , and L. Dong . 2025. “Target Site–Resistance Mechanisms to Imazamox in Imidazolinone Herbicide‐Resistant Weedy Rice ( *Oryza sativa* f. Spontanea) in China.” Weed Science 73: e78. 10.1017/wsc.2025.10048.

[eva70342-bib-0026] Merotto, A. , I. C. G. R. Goulart , A. L. Nunes , et al. 2016. “Evolutionary and Social Consequences of Introgression of Nontransgenic Herbicide Resistance From Rice to Weedy Rice in Brazil.” Evolutionary Applications 9: 837–846. 10.1111/eva.12387. PMC4947146 27468302

[eva70342-bib-0027] Presotto, A. , F. Hernández , R. B. Vercellino , et al. 2024. “Introgression From Local Cultivars Is a Driver of Agricultural Adaptation in Argentinian Weedy Rice.” Molecular Ecology 33: e17368. 10.1111/mec.17368. 38676602

[eva70342-bib-0028] Qiu, J. , L. Jia , D. Wu , et al. 2020. “Diverse Genetic Mechanisms Underlie Worldwide Convergent Rice Feralization.” Genome Biology 21: 70. 10.1186/s13059-020-01980-x. PMC7098168 32213201

[eva70342-bib-0029] Rosas, J. E. , V. Bonnecarrère , and F. Pérez de Vida . 2014. “One‐Step, Codominant Detection of Imidazolinone Resistance Mutations in Weedy Rice ( *Oryza sativa* L.).” Electronic Journal of Biotechnology 17: 95–101. 10.1016/j.ejbt.2014.02.003.

[eva70342-bib-0030] Roso, A. C. , A. M. Jr , C. A. Delatorre , and V. G. Menezes . 2010. “Regional Scale Distribution of Imidazolinone Herbicide‐Resistant Alleles in Red Rice ( *Oryza sativa* L.) Determined Through SNP Markers.” Field Crops Research 119: 175–182. 10.1016/j.fcr.2010.07.006.

[eva70342-bib-0031] Rudell, E. C. , L. Cutti , G. M. Turra , et al. 2025. “Variability and Spatial Distribution of ALS‐Inhibitor Resistance Mechanisms in Brazilian *Echinochloa crus‐galli* .” Plant Physiology and Biochemistry 228: 110237. 10.1016/j.plaphy.2025.110237. 40684739

[eva70342-bib-0032] Shivrain, V. K. , N. R. Burgos , D. R. Gealy , M. A. Sales , and K. L. Smith . 2009. “Gene Flow From Weedy Red Rice ( *Oryza sativa* L.) to Cultivated Rice and Fitness of Hybrids.” Pest Management Science 65: 1124–1129. 10.1002/ps.1802. 19530257

[eva70342-bib-0033] Singh, V. , S. Singh , H. Black , et al. 2017. “Introgression of Clearfield Rice Crop Traits Into Weedy Red Rice Outcrosses.” Field Crops Research 207: 13–23. 10.1016/j.fcr.2017.03.004.

[eva70342-bib-0034] Sudianto, E. , S. Beng‐Kah , N. Ting‐Xiang , N. E. Saldain , R. C. Scott , and N. R. Burgos . 2013. “Clearfield Rice: Its Development, Success, and Key Challenges on a Global Perspective.” Crop Protection 49: 40–51. 10.1016/j.cropro.2013.02.013.

[eva70342-bib-0035] Sun, Y. , X. Zhang , C. Wu , et al. 2016. “Engineering Herbicide‐Resistant Rice Plants Through CRISPR/Cas9‐Mediated Homologous Recombination of Acetolactate Synthase.” Molecular Plant 9: 628–631. 10.1016/j.molp.2016.01.001. 26768120

[eva70342-bib-0036] Svec, D. , A. Tichopad , V. Novosadova , M. W. Pfaffl , and M. Kubista . 2015. “How Good Is a PCR Efficiency Estimate: Recommendations for Precise and Robust qPCR Efficiency Assessments.” Biomolecular Detection and Quantification 3: 9–16. 10.1016/j.bdq.2015.01.005. PMC4822216 27077029

[eva70342-bib-0037] Turra, G. M. , L. Cutti , P. S. Angonese , et al. 2026. “Baseline Sensitivity and Susceptibility Status of Weedy Rice and Echinochloa spp. to ACCase Inhibitors Before the Introduction of FOP‐Resistant Rice in Brazil.” Advances in Weed Science 44: e020260037. 10.51694/AdvWeedSci/2026;44:0028.

[eva70342-bib-0038] Unan, R. , O. Azapoglu , İ. Deligoz , H. Mennan , and K. Al‐Khatib . 2024. “Gene Flow and Spontaneous Mutations Are Responsible for Imidazolinone Herbicide‐Resistant Weedy Rice ( *Oryza sativa* L.).” Pesticide Biochemistry and Physiology 198: 105746. 10.1016/j.pestbp.2023.105746. 38225089

[eva70342-bib-0039] Vila‐Aiub, M. M. 2019. “Fitness of Herbicide‐Resistant Weeds: Current Knowledge and Implications for Management.” Plants 8: 469. 10.3390/plants8110469. PMC6918315 31683943

[eva70342-bib-0040] Wedger, M. J. , N. Roma‐Burgos , and K. M. Olsen . 2022. “Genomic Revolution of US Weedy Rice in Response to 21st Century Agricultural Technologies.” Communications Biology 5: 1–9. 10.1038/s42003-022-03803-0. PMC9458635 36076028

[eva70342-bib-0041] Yu, Q. , H. Han , M. M. Vila‐Aiub , and S. B. Powles . 2010. “AHAS Herbicide Resistance Endowing Mutations: Effect on AHAS Functionality and Plant Growth.” Journal of Experimental Botany 61: 3925–3934. 10.1093/jxb/erq205. PMC2935867 20627897

[eva70342-bib-0042] Zhao, F. , N. A. Maren , P. Z. Kosentka , et al. 2021. “An Optimized Protocol for Stepwise Optimization of Real‐Time RT‐PCR Analysis.” Horticulture Research 8: 179. 10.1038/s41438-021-00616-w. PMC8325682 34333545

[eva70342-bib-0043] Ziska, L. H. , D. R. Gealy , N. Burgos , et al. 2015. “Weedy (Red) Rice: An Emerging Constraint to Global Rice Production.” In Advances in Agronomy, edited by D. L. Sparks , 181–228. Academic Press. 10.1016/bs.agron.2014.09.003.

