# Supplementary material for: Legacy of Recurrent Gene Flow and Evidence of Independent Evolution of ALS Resistance Mutations in Weedy Rice
Source: Evol Appl. 2026 Oct 27;19(11):e70342. doi: 10.1111/eva.70342 (PMC13714446; doi:10.1111/eva.70342)
Supplement: Supplementary file 1 [file EVA-19-e70342-s001.docx]

Supplementary material

ARTICLE TITLE: Legacy of recurrent gene flow and evidence of independent evolution of ALS resistance mutations in weedy rice

Supplementary Figure 1. Validation of SNAP molecular markers for mutations not related to imidazoline-resistant rice cultivars. Melt curve plots with single peaks for weedy rice accessions and the three SNAP primers pairs for the new ALS substitutions. IRGA417 was used as negative control and NTC is the non-template control. Tm: melt temperature.

Supplementary Figure 2. Standard curves used to estimate the SNAP molecular markers efficiency (%) developed for the detection of ALS resistance mutations in weedy rice (A) Ala122Thr (A122T), (B) Ser653Asn (S653N), and (C) Gly654Glu (G654E).

Supplementary Figure 3. Sensitivity and calibration of SNAP molecular markers targeting the ALS resistance mutations Ala122Thr (A, D), Ser653Asn (B, E), and Gly654Glu (C, F). For sensitivity analysis (A-C), resistant and susceptible DNA samples were mixed at defined proportions (1:50, 1:40, 1:30, 1:20, 1:10, and 1:0), and marker performance was evaluated using normalized quantification cycle (ΔCq) values relative to the actin1 reference gene. Values represent the mean ± standard deviation of four technical replicates. Calibration curves (D-F) show the relationship between normalized ΔCq values and the number of resistant plants in composite samples containing up to 10 individuals.

Supplementary Figure 4. Alignment of partial sequences of the *Oryza sativa* ALS gene with the reference sequence DQ516980.1, generated from sequencing of the two regions of the *ALS* gene (primers AR6 and AR6, Table 1). Nucleotides highlighted in yellow represent nonsynonymous mutations.

Supplementary Table 1. Parameter estimates of the three-parameter log-logistic model (LL.3) describing the dose-response relationship for fresh biomass of weedy rice accessions carrying different mutations treated with imazethapyr, penoxsulam, nicosulfuron, and cloransulam-methyl.

| Genotype | b | | d | | GR_50_ (e) | | SE (GR_50_) | RI | |
| --- | --- | --- | --- | --- | --- | --- | --- | --- | --- |
| Imazethapyr | | | | | | | | | |
| Wild type | 2.537 | * | 15.43 | * | 4.60 | * | 1.02 | 1.00 |  |
| Ala122Thr | 1.608 | * | 15.60 | * | 641.31 | * | 67.40 | 139.5 | * |
| Ser653Asn | 2.716 | * | 15.44 | * | 397.64 | * | 28.98 | 86.5 | * |
| Gly654Glu | 1.184 | * | 18.20 | * | 48.57 | * | 6.65 | 10.6 | * |
| Ala205Val | 4.471 | ns | 7.69 | * | 359.66 | * | 36.57 | 78.2 | * |
| Trp574Leu | Lack of fit | | |  |  |  |  |  |  |
| Ser653Thr | 2.365 | *** | 14.90 | * | 280.07 | * | 30.71 | 61.0 | * |
| Penoxsulam | | | | | | | | | |
| Wild type | 0.712 | * | 4.51 | * | 250.50 | ns | 177.82 | 1.00 |  |
| Ala122Thr | 0.673 | * | 5.15 | * | 518.60 | ns | 351.60 | 2.07 | ns |
| Ser653Asn | 0.585 | ns | 2.41 | * | 251.50 | ns | 454.06 | 1.00 | ns |
| Gly654Glu | 0.779 | * | 5.11 | * | 239.85 | ns | 150.18 | 0.96 | ns |
| Ala205Val | 0.856 | ns | 3.29 | * | 631.27 | ns | 654.92 | 2.52 | ns |
| Trp574Leu | Lack of fit | | |  |  |  |  |  |  |
| Ser653Thr | 0.781 | * | 7.62 | * | 265.48 | * | 116.73 | 1.06 | ns |
| Nicosulfuron | | | | | | | | | |
| Wild type | 1.156 | * | 19.12 | * | 9.06 | * | 1.21 | 1.00 |  |
| Ala122Thr | 1.006 | * | 17.70 | * | 9.70 | * | 1.59 | 1.07 | ns |
| Ser653Asn | 0.902 | * | 16.54 | * | 28.65 | * | 5.80 | 3.16 | * |
| Gly654Glu | 1.697 | * | 16.62 | * | 24.15 | * | 2.83 | 2.66 | * |
| Ala205Val | 2.113 | * | 15.54 | * | 18.98 | * | 1.78 | 2.10 | * |
| Trp574Leu | Lack of fit | | |  |  |  |  |  |  |
| Ser653Thr | 0.905 | * | 18.08 | * | 21.67 | * | 3.93 | 2.39 | * |
| Cloransulam-methyl | | | | | | | | | |
| Wild type | 0.722 | * | 15.17 | * | 41.23 | * | 9.61 | 1.00 |  |
| Ala122Thr | 0.638 | * | 11.76 | * | 6.39 | * | 3.14 | 0.15 | * |
| Ser653Asn | 0.558 | * | 12.18 | * | 11.40 | ns | 5.81 | 0.28 | * |
| Gly654Glu | 0.475 | * | 15.73 | * | 21.12 | * | 10.10 | 0.51 | ns |
| Ala205Val | 0.402 | * | 15.56 | * | 96.34 | ns | 49.59 | 2.34 | ns |
| Trp574Leu | Lack of fit | | |  |  |  |  |  |  |
| Ser653Thr | 0.751 | * | 15.26 | * | 36.14 | * | 7.88 | 0.88 | ns |

The table includes estimates of the slope around the inflection point (b), upper limit (d), and the effective dose causing 50% growth reduction (e, GR_50_). SE: standard error (α = 0.05). RI: resistance index. *Significant parameter at the 5% level. ns: not significant.

Supplementary Table 2. Weedy rice accessions information and control efficacy (%) after imazapyr + imazapic treatment (n=3).

| ID | Original name | Sampling year | Control efficinecy (%) | | Marker amplified |
| --- | --- | --- | --- | --- | --- |
|  |  |  |  |  |  |
| 1 | AD2401 | 2023/24 | 0 | E^a^ | A122T |
| 2 | 5 | 2024/25 | 15 | D | A122T |
| 3 | 317 | 2020/21 | 0 | E | A122T |
| 4 | 318 | 2020/21 | 0 | E | A122T |
| 5 | 318.2 | 2020/21 | 0 | E | A122T |
| 6 | 321 | 2020/21 | 0 | E | A122T |
| 7 | 323 | 2020/21 | 0 | E | A122T |
| 8 | 324 | 2020/21 | 0 | E | A122T |
| 9 | 326 | 2020/21 | 0 | E | A122T |
| 10 | 327 | 2020/21 | 0 | E | A122T |
| 11 | 328 | 2020/21 | 0 | E | A122T |
| 12 | 320 | 2020/21 | 0 | E | G654E |
| 13 | 325 | 2020/21 | 63.3 | B | G654E |
| 14 | 325.2 | 2020/21 | 70 | B | G654E |
| 15 | 317.2 | 2020/21 | 0 | E | S653N |
| 16 | 319 | 2020/21 | 0 | E | S653N |
| 17 | AD2411 | 2023/24 | 0 | E | A122T |
| 18 | AD2407 | 2023/24 | 0 | E | A122T |
| 19 | 226 | 2020/21 | 0 | E | A122T |
| 20 | 226.2 | 2020/21 | 0 | E | A122T |
| 21 | 228 | 2020/21 | 0 | E | A122T |
| 22 | 228.2 | 2020/21 | 0 | E | A122T |
| 23 | 234 | 2020/21 | 0 | E | A122T |
| 24 | 234.2 | 2020/21 | 0 | E | A122T |
| 25 | 245 | 2020/21 | 0 | E | A122T |
| 26 | 245.2 | 2020/21 | 0 | E | A122T |
| 27 | 232.2 | 2020/21 | 53.3 | C | G654E |
| 28 | 238 | 2020/21 | 63.3 | B | G654E |
| 29 | 238.2 | 2020/21 | 0 | E | G654E |
| 30 | 241 | 2020/21 | 33.3 | D | G654E |
| 31 | 232 | 2020/21 | 0 | E | W574L |
| 32 | 241.2 | 2020/21 | 0 | E | A205V |
| 33 | PC65 | 2018/19 | 100 | A | None |
| 34 | 356 | 2020/21 | 0 | E | A122T |
| 35 | 359 | 2020/21 | 0 | E | A122T |
| 36 | 361 | 2020/21 | 0 | E | A122T |
| 37 | 362 | 2020/21 | 0 | E | A122T |
| 38 | 365 | 2020/21 | 0 | E | A122T |
| 39 | 366 | 2020/21 | 0 | E | A122T |
| 40 | 368 | 2020/21 | 0 | E | A122T |
| 41 | 369 | 2020/21 | 0 | E | A122T |
| 42 | 369.2 | 2020/21 | 0 | E | A122T |
| 43 | AD2302 | 2022/23 | 0 | E | A122T |
| 44 | AD2303.2 | 2022/23 | 0 | E | A122T |
| 45 | 372 | 2020/21 | 0 | E | S653N |
| 46 | 373 | 2020/21 | 0 | E | S653N |
| 47 | AD2303 | 2022/23 | 0 | E | S653N |
| 48 | 254 | 2020/21 | 43.3 | C | G654E |
| 49 | 254.2 | 2020/21 | 60 | B | G654E |
| 50 | 398 | 2020/21 | 0 | E | A122T |
| 51 | 400 | 2020/21 | 0 | E | A122T |
| 52 | 401 | 2020/21 | 0 | E | A122T |
| 53 | 405 | 2020/21 | 0 | E | A122T |
| 54 | 407 | 2020/21 | 0 | E | A122T |
| 55 | 412 | 2020/21 | 0 | E | A122T |
| 56 | 414_2 | 2020/21 | 0 | E | A122T |
| 57 | 403 | 2020/21 | 0 | E | A205V |
| 58 | 410 | 2020/21 | 100 | A | None |
| 59 | 414 | 2020/21 | 20 | D | A205V |
| 60 | 395 | 2020/21 | 0 | E | S653N |
| 61 | 207 | 2020/21 | 0 | E | A122T |
| 62 | 207.2 | 2020/21 | 0 | E | A122T |
| 63 | 215 | 2020/21 | 0 | E | A122T |
| 64 | 215.2 | 2020/21 | 0 | E | A122T |
| 65 | 225 | 2020/21 | 0 | E | A122T |
| 66 | 225.2 | 2020/21 | 0 | E | A122T |
| 67 | 211 | 2020/21 | 66.7 | B | G654E |
| 68 | 220 | 2020/21 | 100 | A | None |
| 69 | 220.2 | 2020/21 | 100 | A | None |
| 70 | 206 | 2020/21 | 0 | E | S653N |
| 71 | 206.2 | 2020/21 | 0 | E | S653N |
| 72 | AD2307 | 2022/23 | 100 | A | None |
| 73 | AD2306 | 2022/23 | 0 | E | A122T |
| 74 | 174 | 2020/21 | 0 | E | A122T |
| 75 | 174.2 | 2020/21 | 0 | E | A122T |
| 76 | 6 | 2024/25 | 10 | D | A122T |
| 77 | AD2404 | 2023/24 | 38 | C | G654E |
| 78 | 1 | 2024/25 | 70 | B | S653N |
| 79 | 2 | 2024/25 | 15 | D | S653N |
| 80 | 3 | 2024/25 | 15 | D | S653N |
| 81 | 4 | 2024/25 | 15 | D | S653N |
| 82 | PC52 | 2018/19 | 100 | A | A122T |
| 83 | PC53 | 2018/19 | 0 | E | A122T |
| 84 | PC54 | 2018/19 | 0 | E | A122T |
| 85 | PC55 | 2018/19 | 0 | E | A122T |
| 86 | PC56 | 2018/19 | 0 | E | A122T |
| 87 | AD2408 | 2023/24 | 0 | E | A122T |
| 88 | 417 | 2020/21 | 0 | E | A122T |
| 89 | 426 | 2020/21 | 0 | E | A122T |
| 90 | 427 | 2020/21 | 0 | E | A122T |
| 91 | 429 | 2020/21 | 0 | E | A122T |
| 92 | 429.2 | 2020/21 | 0 | E | A122T |
| 93 | 418 | 2020/21 | 73.3 | B | G654E |
| 94 | 420 | 2020/21 | 66.7 | B | G654E |
| 95 | 421 | 2020/21 | 50 | C | G654E |
| 96 | 423 | 2020/21 | 96.7 | A | None |
| 97 | 424 | 2020/21 | 100 | A | None |
| 98 | 433 | 2020/21 | 0 | E | S653T |
| 99 | 437 | 2020/21 | 95 | A | None |
| 100 | 428 | 2020/21 | 0 | E | S653N |
| 101 | 307 | 2020/21 | 0 | E | A122T |
| 102 | 308 | 2020/21 | 0 | E | A122T |
| 103 | 310 | 2020/21 | 0 | E | A122T |
| 104 | 311 | 2020/21 | 0 | E | A122T |
| 105 | 315 | 2020/21 | 0 | E | A122T |
| 106 | PC44 | 2018/19 | 0 | E | A122T |
| 107 | PC45 | 2018/19 | 0 | E | A122T |
| 108 | PC46 | 2018/19 | 0 | E | A122T |
| 109 | PC57 | 2018/19 | 0 | E | A122T |
| 110 | PC43 | 2018/10 | 100 | A | None |
| 111 | 309 | 2020/21 | 0 | E | S653N |
| 112 | 313 | 2020/21 | 0 | E | S653N |
| 113 | 313.2 | 2020/21 | 0 | E | S653N |
| 114 | 314 | 2020/21 | 0 | E | S653N |
| 115 | 314.2 | 2020/21 | 0 | E | S653N |
| 116 | AD2410 | 2023/24 | 0 | E | A122T |
| 117 | AD2301 | 2022/23 | 0 | E | A122T |
| 118 | AD2406 | 2023/24 | 9 | E | A122T |
| 119 | 8 | 2024/25 | 25 | D | A122T |
| 120 | AD2201 | 2021/22 | 0 | E | A122T and G654E |
| 121 | AD2201.2 | 2021/22 | 33 | D | G654E |
| 122 | 7 | 2024/25 | 75 | B | G654E |
| 123 | AD2405 | 2023/24 | 97 | A | None |
| 124 | 333 | 2020/21 | 0 | E | A122T |
| 125 | 335 | 2020/21 | 0 | E | A122T |
| 126 | 336 | 2020/21 | 0 | E | A122T |
| 127 | 337 | 2020/21 | 0 | E | A122T |
| 128 | 340 | 2020/21 | 0 | E | A122T |
| 129 | 340_2 | 2020/21 | 0 | E | A122T |
| 130 | 341 | 2020/21 | 0 | E | A122T |
| 131 | 348 | 2020/21 | 43.3 | C | G654E |
| 132 | 348.2 | 2020/21 | 56.7 | C | G654E |
| 133 | 350 | 2020/21 | 20 | D | A205V |
| 134 | 351 | 2020/21 | 96.7 | A | None |
| 135 | 343 | 2020/21 | 0 | E | S653N |
| 136 | 344 | 2020/21 | 0 | E | S653N |
| 137 | 346 | 2020/21 | 0 | E | S653N |
| 138 | PC64 | 2018/19 | 0 | E | A122T |
| 139 | 452 | 2020/21 | 0 | E | A122T |
| 140 | 456 | 2020/21 | 0 | E | A122T |
| 141 | 456.2 | 2020/21 | 0 | E | A122T |
| 142 | 445 | 2020/21 | 46.7 | C | G654E |
| 143 | 447 | 2020/21 | 43.3 | C | G654E |
| 144 | 442 | 2020/21 | 20 | D | A205V |
| 145 | 440 | 2020/21 | 0 | E | S653N |
| 146 | 440.2 | 2020/21 | 0 | E | S653N |
| 147 | 441 | 2020/21 | 0 | E | S653N |
| 148 | 449 | 2020/21 | 0 | E | S653N |
| 149 | 451 | 2020/21 | 0 | E | S653N |
| 150 | 153 | 2020/21 | 0 | E | A122T |
| 151 | 153.2 | 2020/21 | 0 | E | A122T |
| 152 | 162 | 2020/21 | 0 | E | A122T |
| 153 | 162.2 | 2020/21 | 0 | E | A122T |
| 154 | 162.3 | 2020/21 | 0 | E | A122T |
| 155 | 163 | 2020/21 | 0 | E | A122T |
| 156 | 163.2 | 2020/21 | 0 | E | A122T |
| 157 | 203 | 2020/21 | 0 | E | A122T |
| 158 | 205 | 2020/21 | 0 | E | A122T |
| 159 | 205.2 | 2020/21 | 0 | E | A122T |
| 160 | 202.2 | 2020/21 | 0 | E | A122T and G654E |
| 161 | 289 | 2020/21 | 0 | E | A122T |
| 162 | 292 | 2020/21 | 0 | E | A122T |
| 163 | 296 | 2020/21 | 76.7 | B | G654E |
| 164 | 298 | 2020/21 | 63.3 | B | G654E |
| 165 | 299 | 2020/21 | 76.7 | B | G654E |
| 166 | 300 | 2020/21 | 91.7 | A | G654E |
| 167 | 302 | 2020/21 | 66.7 | B | G654E |
| 168 | 302.2 | 2020/21 | 73.3 | B | G654E |
| 169 | 305 | 2020/21 | 56.7 | C | G654E |
| 170 | 280.2 | 2020/21 | 72.5 | B | G654E |
| 171 | 280.3 | 2020/21 | 76.7 | B | G654E |
| 172 | 284.2 | 2020/21 | 0 | E | A205V |
| 173 | 286 | 2020/21 | 0 | E | A205V |
| 174 | PC38 | 2018/19 | 0 | E | A122T |
| 175 | PC39 | 2018/19 | 0 | E | A122T |
| 176 | PC40 | 2018/19 | 0 | E | A122T |
| 177 | PC41 | 2018/19 | 0 | E | A122T |
| 178 | PC42 | 2018/19 | 0 | E | A122T |
| 179 | PC63 | 2018/19 | 0 | E | S653N |
| 180 | 380 | 2020/21 | 0 | E | A122T |
| 181 | 391 | 2020/21 | 0 | E | A122T |
| 182 | 393 | 2020/21 | 0 | E | A122T |
| 183 | 385 | 2020/21 | 38.3 | C | G654E |
| 184 | 377 | 2020/21 | 0 | E | A205V |
| 185 | 383 | 2020/21 | 0 | E | A205V |
| 186 | 375 | 2020/21 | 0 | E | S653N |
| 187 | 378 | 2020/21 | 0 | E | S653N |
| 188 | 387 | 2020/21 | 0 | E | S653N |
| 189 | 388 | 2020/21 | 0 | E | S653N |
| 190 | 388.2 | 2020/21 | 33.3 | D | S653N |
| 191 | PC66 | 2018/19 | 0 | E | A122T |
| 192 | PC69 | 2018/19 | 0 | E | A122T |
| 193 | PC70 | 2018/19 | 0 | E | A122T |
| 194 | AD2409 | 2024/23 | 0 | E | A122T |
| 195 | AD2304 | 2022/23 | 0 | E | A122T and G654E |
| 196 | AD2305 | 2022/23 | 39.7 | C | G654E |
| 197 | PC68 | 2018/19 | 100 | A | None |
| 198 | PC71 | 2018/19 | 100 | A | None |
| 199 | AD2402 | 2024/23 | 0 | E | A122T |
| 200 | AD2403 | 2024/23 | 100 | A | None |

^a^Means followed by the same letter are statistically equal according to the Skott-Knott test (5%)

Supplementary Table 3. Sequence, annealing temperature (Ta), melting temperature (Tm), size of the expected fragment in base pairs (bp), number of cycles required for correct genotyping, and the covered position in the *ALS* gene for the SNAP markers and sequencing primers.

| Primer name | Sequence (5’-3’) | Ta (ºC) | Cycles | Tm (ºC) | Fragment (bp) | Covered position^d^ |
| --- | --- | --- | --- | --- | --- | --- |
| L574_F | TTGGGTATGGTGGTGCAGT**T** | 56 | 40 | 77.1 | 65 | Trp574Leu |
| W574L_R | CCCAAGTATGTATGCGCCCT |  |  |  |  |  |
| A205_F | CGCATGATCGGCACCGGCG**T** | 66 | 35 | 87.6 | 190 | Ala205Val |
| V205V_R | TCTGCTGCTGGATGTCCTTG |  |  |  |  |  |
| S653T_F | GTGCTGCCTATGATCCCAG**C** | 64 | 35 | 80.9 | 62 | Ser653Thr |
| S653_R | CTGCCATCACCATCCAGGAT |  |  |  |  |  |

Bold letter indicates the SNP and underlined letter indicates the SNAP for allele-specific qPCR.

^d^ Based on *ALS* gene sequence from *Arabidopsis thaliana*
